# Role of Tissue and Systemic Hypoxia in Obesity and Type 2 Diabetes

**DOI:** 10.1155/2016/1527852

**Published:** 2016-06-21

**Authors:** Lei Xi, Chin-Moi Chow, Xingxing Kong

**Affiliations:** ^1^Pauley Heart Center, Division of Cardiology, Virginia Commonwealth University, Richmond, VA 23298-0204, USA; ^2^Department of Sports Medicine, Chengdu Sport University, Chengdu 610041, China; ^3^Delta Sleep Research Unit, Exercise Health and Performance Group, Faculty of Health Sciences, The University of Sydney, Sydney, NSW 2141, Australia; ^4^Division of Endocrinology, Beth Israel Deaconess Medical Center, Harvard Medical School, Boston, MA 02215, USA

Human lifestyle in most modern and developing societies has dramatically changed over past decades. Physical inactivity along with unrestricted access to calorie dense foods has established an “obesogenic” environment and contributed to a serious epidemic of obesity and type 2 diabetes (T2D), associated with increased morbidity and mortality. In 2005 a population-based study conducted by Reichmuth et al. of University of Wisconsin with a cross-sectional and longitudinal analysis identified that among 1387 participants the odds ratio for T2D with an apnea-hypopnea index (AHI) > 15 versus an AHI < 5 was 2.30 (1.28–4.11; *p* < 0.01) after adjustment for age, sex, and body habitus [1]. Therefore it has been assumed that intermittent hypoxic periods associated with obstructive sleep apnea (OSA) may play a pathogenic role in inducing insulin resistance and T2D. At organ/tissue levels, in 2007–2009 Ye and colleagues first proposed a central role played by adipose tissue hypoxia resulting from adipocyte expansion in promoting chronic inflammation, adiponectin reduction, adipocyte dysfunction, and death in obese individuals [2, 3]. This group of researchers later identified the mediator roles played by hypoxia inducible factor 1*α* (HIF-1*α*) [4] and other hypoxia-triggered signaling mechanisms that may promote free fatty acid release and inhibit glucose uptake in adipocytes by inhibition of the insulin-signaling pathway and induction of cell death [5].

Furthermore, at the whole-body level, systemic nocturnal intermittent hypoxia was shown to be associated with increased risk of developing T2D in middle-aged men [6]. Most recently, another elegant study examined 601 participants who were originally enrolled into the Wisconsin Sleep Cohort around 18 years ago and found that hypoxia may be a stimulus for cardiac hypertrophy in individuals with OSA [7]. This study showed that the decade-long occurrence of OSA was associated independently with decreasing left ventricular systolic function and with reduced right ventricular function. Echocardiographic measures of adverse cardiac remodeling were strongly associated with OSA but were confounded by obesity. Nevertheless, it remains elusive how systemic versus local tissue hypoxia affects the key pathophysiological processes of obesity and T2D, such as metabolic imbalance, inflammation, dysglycemia, and insulin resistance. Conversely, the changes resulting from T2D may also alter the adaptation ability or resistance of an organ against tissue injuries caused by hypoxia or ischemia and in turn affect the progression and outcome of many chronic diseases. For example, the studies from our and other groups revealed that obese and/or T2D animals were refractory to ischemic postconditioning, a promising cardioprotective modality against myocardial infarction [8–10].

Under this context, our primary goal for guest-editing this special issue is to provide a platform for insightful discussions and exchanges of divergent ideas concerning systemic and local hypoxia as trigger or treatment (in some cases) of adipose tissue dysfunction and other organ injuries in obesity and T2D, to invite and showcase the cutting edge original research articles and reviews focusing on the impact of continuous or intermittent systemic hypoxia (e.g., altitude training and OSA), as well as localized tissue hypoxia (in adipose and other types of tissues), on the pathogenesis, progression, and possible novel treatments of obesity and T2D. New revelation of brown adipose tissue activity in adult humans has stimulated vigorous investigations on how it can serve as a prevention and treatment target for obesity and insulin resistance; a particular emphasis is to analyze the role of brown adipose tissue in whole-body energy homeostasis and substrate metabolism under normal and hypoxic conditions.

Out of a dozen of submitted manuscripts in response to our call-for-papers, seven papers authored by 35 biomedical researchers or physician scientists from China, Hong Kong, Slovenia, United Kingdom, and United States have been selected through a rigorous peer-review process and finally included in this special issue. The following are some highlights of these accepted works.

Most relevant to the main theme, the review article by R. W. A. Mackenzie and P. Watt has provided insights at the molecular and whole-body levels on the mechanisms surrounding glucose disposal and insulin resistance with systemic exposure to chronic hypoxia. These authors thoughtfully discussed the complex and paradoxically opposing effects of hypoxia on the development of insulin resistance. On the one hand, hypoxia may induce insulin resistance either via the direct action on insulin receptor substrate and protein kinase B/Akt or indirectly through adipose tissue expansion and systemic inflammation. Yet hypoxia may also promote glucose transport via insulin-dependent mechanisms largely reliant on AMP-activated protein kinase (AMPK) and hypoxic exposure could improve glucose control in T2D. The authors' provocative conclusion is that hypoxia may decrease insulin signaling but may not induce whole-body insulin resistance.

Another focus of this special issue has been on adipose tissue, a biologically active organ that is vital for lipid storage, energy homeostasis, and whole-body insulin sensitivity [11]. Three articles have addressed this important topic. P. Zhou et al. explored the effects of inhibitor of DNA binding 2 gene (Id2) deficiency in the regulation of core body temperature over the circadian cycle and the impact of Id2 deficiency on genes involved in insulin signaling and adipogenesis in brown adipose tissue. Their findings lend support for Id2 as an important coordinator of energy homeostasis including insulin signaling, adipogenic programing, and thermoregulation. In addition, at the adipocyte level, J. Sun et al. demonstrated that free fatty acids activated the renin-angiotensin system in mouse 3T3-L1 adipocytes through a nuclear factor-kappa B-dependent signaling pathway, which shed light on the molecular basis for a vicious cycle between obesity and adipose tissue inflammation. Importantly, the authors identified the signaling pathways of activation of local adipose RAS in preadipocyte. They found that free fatty acid (palmitic acid, PA) could upregulate the expression of angiotensinogen and angiotensin type 1 receptor and also stimulate the secretion of angiotensin II in 3T3-L1 adipocytes. Moreover, the activation of renin-angiotensin system in 3T3-L1 adipocytes was inhibited when Toll-like receptor 4 (TLR4) signaling pathway was blocked by TLR4 inhibitor or NF-*κ*B inhibitor, suggesting involvement of the PA/TLR4/NF-*κ*B signaling pathway in regulating the local renin-angiotensin system in adipose tissue. More relevant to the hypoxia-related problems, H. H. Chowdhury et al. provided evidence that hypoxia alters expression of dipeptidyl peptidase 4 (DPP4) and induces developmental remodeling of human preadipocytes. The authors indicated that hypoxia strongly inhibits DPP4 protease activity and insulin-mediated increase in DPP4 suggesting that DPP4 represents an important marker for early detection of insulin resistance.

Two articles in this special issue targeted myocardial ischemia-reperfusion injury, an important pathological condition complicated by obesity and/or T2D. L. Pang et al. thoroughly analyzed the less understood prostaglandin E receptor subtype 4 signaling in the heart, which could profoundly impact ischemia-reperfusion injury and cardiac hypertrophic response in T2D. Metformin, a first-line medication for the treatment of T2D, was reported to be protective against cardiac ischemia-perfusion injury [12]. In this special issue, M. Hu et al. provided further evidence for metformin-induced cytoprotection against hypoxia-reoxygenation injury in H9C2 rat cardiomyocytes under normal and hyperglycemic conditions. The authors demonstrated that the protection may be via an intracellular signaling mechanism involving AMPK and JNK (Jun NH(2)-terminal kinase). Given this commonly prescribed medication, an understanding of its underlying mechanisms is vital both for clinicians and for patients.

Finally, a review by D. Grinnan et al. presented a framework with epidemiological, clinical, biochemical, and molecular evidence to support a role for diabetes mellitus in the pathogenesis of pulmonary arterial hypertension. The review, in this issue, is both timely and significant in trumpeting the need for future robust research to confirm an association between diabetes mellitus and pulmonary hypertension, to demonstrate diabetes as a disease modifier of pulmonary arterial hypertension, and to investigate the outcome of glycemic control on pulmonary hypertension. From an editorial perspective, this review underscores the need for future investigations in endothelial dysfunction in pulmonary arterial hypertension in patients with diabetes who have comorbid conditions of chronic obstructive pulmonary disease and OSA, since hypoxia experienced in these patient groups has an added vasoconstrictive stress on the pulmonary vasculature [13]. Notably, the interaction between intermittent hypoxia and obesity/T2D was not covered in the current issue. This topic deserves greater attention. Future studies are warranted to investigate this often controversial area, that is, a pathogenic role played by OSA in inducing insulin resistance and T2D in humans [1, 14] versus the exposure to moderate intermittent hypoxia that may serve as a therapeutic means for reducing obesity and T2D [15].

We would like to thank all contributors to this special issue for their participation. We believe that the works presented in this special issue may instigate innovative research and development of new therapeutic drugs for the future treatment of diabetes and diabetes complications. There remain many questions to be answered regarding the role of hypoxia in the pathogenesis and treatment target of obesity and T2D.

*Lei Xi* *Lei Xi* *Chin-Moi Chow* *Chin-Moi Chow* *Xingxing Kong* *Xingxing Kong*
